# Married women with children experience greater intrasexual competition than their male counterparts

**DOI:** 10.1038/s41598-023-31816-0

**Published:** 2023-03-18

**Authors:** Joyce F. Benenson, Henry Markovits

**Affiliations:** 1 Department of Human Evolutionary Biology, Harvard University, Cambridge, 02138 USA; 2 Département de Psychologie, Université du Québec à Montréal, Montréal, H3C 3P8 Canada

**Keywords:** Sexual selection, Social evolution, Human behaviour

## Abstract

Human males are considered to be more competitive than females. However, females must also compete for resources necessary for their own and their offsprings’ survival. Since females use more indirect forms of competition than males, comparing observable forms of competition may be misleading. One critical driver of competition is resource asymmetry. Since competition occurs primarily within sex, reactions to resource asymmetry with same-sex peers should provide an important measure of competitiveness. We asked 596 married participants, 25–45 years of age with at least one child from three different countries to evaluate how same-sex individuals they know would react to a target individual who had a valuable resource that the same-sex individuals did not have. Half the participants evaluated reactions to same-sex targets, while the other half evaluated reactions to other-sex targets. Participants reported that women would react more negatively than men to resource asymmetry with same-sex targets, but not other-sex targets. These results suggest that women may be even more competitive than men in contexts when important resources related to reproductive success are at stake.

## Introduction

Intrasexual competition refers to competition between same-sex individuals for resources, allies or mates that can enhance reproductive success (RS). However, the proximate goals of intrasexual competition and the preferred strategies used to compete with same-sex conspecifics typically vary by sex.

Sexual selection theory has provided a valuable explanation for sex differences in intrasexual competition. According to sexual selection theory, greater variance in RS in the sex that invests less in offspring, typically the male, compared to the sex that invests more, usually the female, produces important differences in the goals and strategies pursued by each sex^1,2^. Specifically, males typically benefit more than females from engaging in competitive behaviors that include direct and potentially harmful forms of competition, including risking injuries and enhancing vulnerability to disease for two major reasons: First, males’ greater variance in RS means many males will not reproduce. Therefore, males face a larger incentive than females to outcompete other males to obtain matings, whereas females are more assured of mating^1^. Second, males incur fewer costs than females from potential harm resulting from direct competition, because females who are more self-protective are far more likely to care for offspring^3,4^. Females who typically care for offspring both as mothers and as grandmothers therefore benefit most from ensuring their survival and health and should thus avoid direct and potentially dangerous forms of competition^4,5^. The result is that males typically have been considered the more competitive sex. Empirical research further demonstrates that across the animal kingdom, competition for dominance status increases RS more for males than for females^2,6,7^.

More recent theoretical and empirical accounts of sexual selection however, have emphasized the importance of female-female competition over resources, allies, and mates to females’ RS^3,8–12^. In female mammals who are responsible for gestation, lactation, and rearing offspring often for years, competition for parenting resources should be highly related to survival and RS of mothers and offspring. In fact, several primatologists postulate that competition for life-sustaining resources may be more frequent and the outcomes more significant for females who must care for offspring throughout their lives than for males who attain matings more sporadically^13,14^ .

Additionally, intrasexual competition takes differing forms in the two sexes, so that while males more frequently engage in direct, conspicuous contests often to enhance their public status or deter others’ mating attempts^8^, females often employ less direct forms of competition^9^, presumably to reduce their chances of sustaining injuries^5^. In terms of competitive tactics, humans fit the mammalian pattern. Human males engage in more direct competition individually or in coalitions than females^15,16^. Cross-culturally and historically, boys and men have entered competitive sport contests with clear winners and losers much more frequently than girls and women have^17–19^. Similarly, in economic games played across diverse cultures boys and men enter contests more than girls and women who prefer to engage in individual efforts^20,21^. Likewise, worldwide boys and men engage in more direct verbal as well as physical contests than girls and women do^15,16,22,23^. Consequently, one well-established descriptor of sex differences in humans is that males are more competitive than females^24,25^.

Nonetheless, women also compete. Women attempt to obtain physical and social resources, including allies and mates, to support themselves and their children using a number of tactics including self-promotion^26^; disguised or indirect competition such as reputation derogation or social exclusion, which do not require directly confronting a rival^15^; and scramble competition in which individuals attempt to do better for themselves, without directly interfering with another’s success^27^.

Previous studies that compared primarily young men’s and women’s self-reports of their own competitiveness in fact have found few sex differences^28–30^. These results offer some preliminary support for the idea that levels of competition between females and males might be more similar than their overtly competitive behavior might suggest.

The difficulty with comparing male and female competitiveness is that while males prefer direct and conspicuous forms of competition, female forms of competition are more difficult to observe. In studies of adults facing important consequences however, several studies suggest that females may be as or more competitive than males. For example, in an economic game, women competing on behalf on their children were just as competitive as men^31^. Additionally, across diverse cultures following the outcome of high level sports tournaments with large monetary payoffs, women exhibit more negative reactions than men^32^. Likewise, a large meta-analysis of laboratory and work environments concluded that women are less likely than men to like same-sex, but not other-sex, individuals who have attained higher positions^33^. Similarly, an American study of > 60,000 individuals showed that women held more negative views than men of their same-sex, but not other-sex, bosses^34^. Nevertheless, some of these forms of intrasexual competition may entail direct contests, which may simply be more dangerous to females than males, hence women’s greater aversion to them^5^.

We reasoned that when actual resources are at stake which could influence survival and RS, asymmetry in resources should provide a major impetus for competition^31,35^. Furthermore, resource asymmetry has been associated with self-reported competitive reactions in females, although the specific pattern of results is mixed. In one study, women predicted that they would be more aggressive when competing in a resource poor context^36^. Similarly women showed increased self-sexualization behavior when income inequality increased, suggesting greater self-promotion^37^. In contrast, women perceive same-sex rivals as more competitive when they are in resource-rich contexts^38^. Finally, there is evidence that resource scarcity affects females’ competitive attitudes, but levels of envy modulate the direction of the effect^39^. Nonetheless, envy of others’ resources is considered a prime motivator of competitiveness^40^.

In summary, theoretically and empirically, there are conflicting reports regarding expected sex differences in competitiveness, especially in humans. Those studies that include real-life consequences however do suggest that human females are at least or more competitive than males with other same-sex adults^31–34^. Thus, we aimed to test this by using a novel approach: asking married adults with children to report on how people they personally know who lack important resources react to someone they have seen who has these resources. Because we are asking participants about familiar people, direct, conspicuous behavior is less important as personal contacts permit access to more private reactions.

Since we intended to directly compare female and male reactions to situations of concrete resource asymmetry, it was important that the resources in question were not themselves biased towards one sex. Thus, we compiled two separate sets of resources broadly defined that are theoretically more important for women (female advantageous) and for men (male advantageous). Traditional sexual selection theory and the empirical research that supports it indicates that mating success is enhanced by attaining higher status which should particularly benefit males^1,2,41^. Parental investment theory and recent research adds the importance of physical and social resources to females who must care for offspring, including finding healthy food, safe territories, and social support^3,9,11,42^. Consequently, we focused on resources that would advantage women in parenting and resources that would advantage men in attracting mates. Table 1 presents the resources and past research supporting the choice of each resources (see Table 1).

**Table 1:** Positive characteristics theoretically advantageous for women and men.

Physical and social resources and physical attractiveness (theoretical advantage to women)	Wording of item	Assets associated with community status (theoretical advantage to men)	Wording of Item
Food is considered the most limiting resource for female mammals who must gestate, lactate, and provide abundant resources for offspring^9,43,44^ and women are more involved than men in food preparation^45,46^	“This (wo)man provides exceptionally healthy and tasty food for her/himself and her/his family”	In non-human primates^47,48^ and human children^49^, objects that move are more attractive to males than females. Further, expensive cars may constitute costly signaling designed to attract mates through showing off status for men more than women^50,51^	“This (wo)man owns a really expensive, high-quality car”
Territory is considered more beneficial to female than male mammals who raise offspring^9,44^ and universally girls and women remain closer to home than boys and men^52,53^	“This (wo)man lives in a beautiful house surrounded by lovely gardens”	In most mammals, strength is critical to fighting ability which is more important for male than female mating success^1,44^, and strength^54^ and throwing ability^55^ are greater in men than women with differences in throwing appearing by early childhood^56^	“This (wo)man has so much physical strength that s/he can lift heavy weights and throw long distances”
Safety from predators and conspecifics as well as environmental pollutants are considered more important for female mammals who care for offspring than males^9,44^ and women are more concerned with safety than men^57^	“This (wo)man lives in a community with excellent services, including top quality police and fire departments and clean water and air”	High energy levels should aid in winning physical fights, and prenatally through adulthood, boys and men have higher energy/activity levels than girls and women^58–60^	“This (wo)man is so energetic that s/he almost never tires”
Physical attractiveness is more important to men than women when selecting a mate^61,62^ so women more than men promote their own physical attractiveness^26^	This (wo)man is strikingly attractive	In most mammals, height is critical to fighting ability which is more important for male than female mating success^1,44^, and men are taller than women worldwide^63^ with greater height associated with higher income^64^	“This (wo)man is very tall and dignified looking”
Prolonged health should be more important for mammalian females’ than males’ reproductive success because offspring require continued care^65^ and North American studies show that women are more concerned than men about their health^66–68^	“This (wo)man is so healthy that s/he almost never feels sick”	Status is often defined as having power over others in both non-humans and humans which women more than men desire in heterosexual partners, making status more important to men^1,6,69^	“This (wo)man holds a lot of power over others at her/his workplace, including making hiring, firing, and salary decisions”
Beginning in infancy, girls are more self-controlled and less impulsive than boys^59^ which presumably reduces their own and their children’s risks	“This (wo)man is so calm, cool, and collected that s/he always maintains her(his) self-control”	Status is often associated with earnings and prestige which women more than men desire in heterosexual partners, making status more important to men^61,62,69^	“This (wo)man earns a lot of money in her/his position at work due to her/his high quality skills”
Universally, women’s parents, particularly their mothers, increase the reproductive success of her children^70^, and both parents invest more in daughters’ than sons’ children^71^	“This (wo)man’s parents help her(him) with childcare and other tasks whenever s/he needs it”	Worldwide, status is often associated with political influence which women more than men desire in heterosexual partners, making community status typically more important to men^69,72,73^	“This (wo)man holds a high-level position in her(his) community’s governance due to her(his) excellent political skills”
While marital bonds occur worldwide, spousal abuse is a universal problem for women^74^ and interparental conflict is harmful for children^75,76^	“This (wo)man and her husband (his wife) get along really well and support one another”		“This (wo)man is highly influential in her/his community as others follow what s/he says and does”
Women worldwide desire high status men^61,62^	“This woman’s husband (man’s wife) is highly successful at his/her job”	In hunter-gatherer societies, brothers appear more important to men than siblings of either sex are to women^77^	“This (wo)man has sisters and brothers who help and support her/him”
Universally, girls and women interact with fewer same-sex peers at a time than boys and men^78–81^	“This (wo)man has several very close, longtime, same-sex friends”	Universally, boys and men spend more time in group activities with unrelated same-sex peers than girls and^73,80,81^ women do, suggesting that male groups are more important than female groups^79^. Cooperative group activities may also provide practice for intergroup aggression^81,82^	“This (wo)man is a member of a group of (wo)men in the community who regularly get together to enjoy a shared activity”
In the USA and Europe, women are more invested in neighbors than men are^83,84^. Universally neighbors likely are more important to women than men because women remain closer to home^52,53^	“This (wo)man and her/his neighbors help one another out when they need a favor”	Men exhibit more positive views of higher-status same-sex peers than women do^33,34^ which may be due to men’s greater comfort with hierarchical groups^81^	“One of this (wo)man’s oldest friends holds a position high up in the government”

The advantage of asking people to report about familiar others is that it avoids the difficulties in observing women’s less conspicuous competitive tactics as well as the pitfalls inherent in self-reports. A major problem with self-reports is the ubiquity of self-deception^85^. Empirical evidence consistently shows that self-reports do not correspond well to actual behavioral patterns^86^. An alternative and more accurate method consists of asking for others’ reports based on their own experiences. Evidence demonstrates that individuals develop accurate models of others’ behavioral reactions^87,88^. Consequently, we asked participants to predict based on their own experiences how most familiar same-sex individuals would react towards a target who possessed a valuable resource or characteristic that the individuals did not possess.

We also included only adults who would themselves understand the importance of resource asymmetry and have access to others like them with the same experiences. Therefore, we specifically included married participants aged 25–45 years with children in order to maximize the ecological validity of our study which was designed to examine resource competition during the critical childbearing and reproductive years in line with parental investment theory^89^. Importantly, unlike most mammals, human males significantly invest in their offspring particularly when they are coupled^90,91^. If males are simply more competitive than females, then they should react more negatively than females to a relative resource deficit. However, despite human male investment in offspring, parental investment theory suggests that females should be more competitive than males in a context in which there are offspring^3^.

To examine the importance of each resource or characteristic to women versus men, we recruited a total of 596 participant-observers online from three different countries (UK, USA, SA). Participants were asked to report only about the reactions of familiar same-sex individuals to 22 different targets, each possessing a different resource or positive characteristic that should be beneficial to parenting or mating. The targets were all in their 30s and married with children. Given that universal sex-segregation of interactions begins in middle childhood^92^ and continues with the cross-cultural sexual division of labor^93^, same-sex individuals should have greater access to information about their own sex, particularly those similar in age and familial status.

In Study 1, we analyzed reports of women’s and men’s reactions to same-sex targets. In Study 2, to control for possible sex biases in valence of responses, we analyzed reports of women’s and men’s reactions to other-sex targets. Participants always described how most individuals of their own sex would react to either a same-sex (Study 1) or other-sex (Study 2) target in his/her 30s who was married and had children.

### Statistical analyses

For both studies, we transformed responses into a dichotomous variable with 1 assigned for negative responses, and 0 otherwise. To examine how sex of participant/observed individuals and sex of advantageous characteristic varied, a general linear mixed model (GLMM) using the package glmer in R with emmeans for contrast analyses was conducted on negative responses (0, 1) using a binomial analysis with a logit link with sex of characteristic type (female advantageous versus male advantageous) as a repeated measure, sex of participant-observer as an independent variable, country as a random effect, and age of participant-observer and age of oldest child as covariates. We also replicated the analyses using the full response scale, which gave the same pattern of results; these are reported in the SOM.

## Results

**Figure 1:**
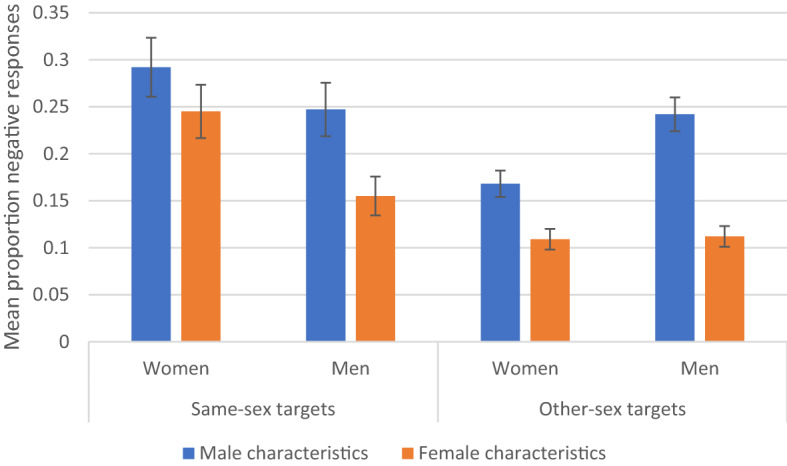
Mean proportion (± SE) of negative responses reported by female and male participants for observed women and men for same-sex (left side) and other-sex (right side) targets for male and female positive characteristics.

For Study 1 which examined reactions to same-sex targets who possessed a positive characteristic that most same-sex individuals did not have, results yielded significant effects of sex of participant-observer, *X*^2^(1) = 155.76, *p* < 0.0001, sex of characteristic type, *X*^2^(1) = 24.52, *p* < 0.0001, and their interaction, *X*^2^(1) = 5.32, *p* = 0.021. No effects of age of participant-observer nor of age of oldest child were found. Significantly more female participant-observers reported that women lacking a characteristic would feel negatively towards women with the characteristic (EMM = 0.274, SE = 0.027, 95% CI [0.222, 0.326]) than male participant-observers reported for men’s reactions towards a man possessing the characteristic (EMM = 0.130, SE = 0.016, 95% CI [0.099, 0.161]). Additionally, more participant-observers reported negative reactions for male advantageous characteristics (EMM = 0.230, SE = 0.029, 95% CI [0.213, 0.326]) than for female advantageous characteristics (EMM = 0.174, SE = 0.019, 95% CI [0.137, 0.211) for both men *and* women. Contrast analyses on the interaction showed that reports of the difference between women’s and men’s negative reactions were significant both for male advantageous characteristics (Male: (EMM = 0.162, SE = 0.019, 95% CI [0.122, 0.202]) Female: (EMM = 0.297, SE = 0.029, 95%, CI [0.241, 0.354]), *z* = 7.256, *p* < 0.0001, and for female advantageous characteristics (Male: (EMM = 0.098, SE = 0.014, 95% CI [0.070, 0.126]), Female: (EMM = 0.251, SE = 0.026, 95%, CI [0.199, 0.302]), *z* = 8.104, *p* < 0.0001, although the relative difference was greater for female-based characteristics as shown on the left in Fig. 1 (see Fig. 1).

A more detailed analysis of participants-observers’ reports of negative evaluations of reactions of same-sex individuals were conducted for each positive characteristic as displayed in Table 2 (see Table 2). As shown in the table, none of the 22 positive characteristics possessed by a same-sex target was reported to elicit significantly more negative evaluations by male participants reporting about men compared with female participants reporting about women. In contrast, significantly more female than male participant-observers reported that women would feel more negatively than men on 10 of the 22 characteristics as displayed in Table 2. In order of descending order of total negative reactions, significantly more female than male participant-observers reported that women would feel more negatively towards another same-sex individual with a powerful position at work, who is physically attractive, has helpful parents, is an influential member of the community, is highly energetic, experiences excellent health, lives in a beautiful house with lovely gardens, gets along with a supportive spouse, is cool, calm, and collected, and serves high quality food. A further eight characteristics elicited non-significantly more negative reactions by female participants reporting about women than male participants reporting about men, whereas four characteristics elicited non-significantly more negative reactions by male participants reporting about men than female participants reporting about women.

**Table 2:** Percentage of female and male participants reporting negative reactions by women and men respectively to each characteristic in descending order of totals.

Characteristic theorized to be more important to Women (W) or Men (M)	Female participants (n = 150)		Male participants (n = 149)		*X*^2^(1)	*p*
	%	(n)	%	(n)		
Expensive car (M)	53.3	(80)	47.0	(70)	1.21	.272
Powerful at work (M)	44.7	(67)	33.6	(50)	3.87	.049
Physically attractive (W)	44.0	(66)	31.5	(47)	4.93	.026
Friend in government^a^ (M)	30.7	(46)	39.6	(59)	2.62	.11
Helpful parents (W)	38.0	(57)	24.2	(36)	6.68	.01
High-level position in community^a^ (M)	28.7	(43)	29.5	(44)	.027	.869
Influential member of community (M)	34.0	(51)	19.5	(29)	8.06	.005
Highly energetic (M)	34.7	(52)	18.8	(28)	9.61	.002
Excellent health (W)	34.7	(52)	18.8	(28)	9.61	.002
Successful spouse (W)	29.3	(44)	20.8	(31)	2.89	.089
Tall and dignified^a^ (M)	24.0	(36)	24.2	(36)	.001	.974
Beautiful house and grounds (W)	28.7	(43)	17.4	(26)	5.30	.021
Highly paid at work (M)	26.7	(40)	18.8	(28)	2.64	.10
Physically strong (M)	20.7	(31)	15.4	(23)	1.38	.24
Safe community with excellent services (W)	18.0	(27)	17.4	(26)	.016	.901
Supportive siblings^a^ (M)	14.7	(22)	18.8	(28)	.913	.339
Longstanding, close, same-sex friends (W)	18.7	(28)	14.1	(21)	1.14	.286
Gets along with supportive spouse (W)	20.0	(30)	7.4	(11)	10.06	.002
Calm, cool, and collected (W)	18.7	(28)	8.1	(12)	7.27	.007
High quality food (W)	17.3	(26)	8.7	(13)	4.88	.027
Shared activity with group (M)	12.0	(18)	8.7	(13)	.863	.353
Helpful neighbors (W)	5.3	(8)	4.0	(6)	.286	.593

^a^More men than women thought this characteristic would be evaluated negatively by same-sex peers.

To examine whether the relative ordering of the different characteristics differed for women and men, the 22 characteristics were ranked in descending order (determined by total number of negative evaluations) separately for both female and male participant-observers. The rank ordering for females and males was highly similar, *r*(20) = 0.82, *p* < 0.0001. For example, according to participants, a same-sex individual who owned an expensive car would be evaluated most negatively by other women and other men without this kind of car, and an individual same-sex individual having helpful neighbors would be evaluated least negatively by other women and men without helpful neighbors. Likewise, a physically attractive target individual while viewed significantly more negatively by women according to female participant-observers than by men according to male participant-observers, nevertheless elicits some of the highest number of negative reactions by both sexes.

Results show that participants report that women feel more negatively than their male counterparts towards same-sex peers with families across a variety of resources. It is possible however that female participants and the women they know simply engage in more negative evaluations than male participants and the men they observe. To examine this possibility, we examined responses to other-sex targets in Study 2.

Results from Study 2 yielded significant effects of sex of participant-observer, *X*^2^(1) = 67.62, *p* < 0.0001, sex of characteristic type, *X*^2^(1) = 108.35, *p* < 0.0001, and age, *X*^2^(1) = 7.30, *p* = 0.007. In marked contrast to Study 1, significantly *fewer* female participant-observers reported that women lacking a characteristic would feel negatively towards men with the characteristic (EMM = 0.099, SE = 0.010, 95% CI [0.079, 0.119]) than male participant-observers reported for men’s reactions towards women possessing the characteristic (EMM = 0.176, SE = 0.015, 95% CI [0.147, 0.206]), as displayed on the right of Fig. 1. As in Study 1, more participant-observers reported negative reactions for male advantageous characteristics (EMM = 0.184, SE = 0.016, 95% CI [0.154, 0.215]) than for female advantageous characteristics (EMM = 0.091, SE = 0.009, 95% CI [0.072, 0.109]). Finally, there was a significant, but slight increase in negative evaluations with age, b = 0.025.

As a final analysis, we combined the results of both studies, and performed a general linear mixed model (GLMM) on negative responses (0, 1) using a binomial analysis with a logit link with sex of characteristic type (female advantageous versus male advantageous) as a repeated measure, sex of participant-observer and condition (same-sex versus other-sex target) as independent variables, with country as a random effect, and age as a covariate. Results yielded significant effects of sex of participant-observer, *X*^2^(1) = 4.12, *p* < 0.042, sex of characteristic type, *X*^2^(1) = 134.34, *p* < 0.0001, condition, *X*^2^(1) = 119.70, *p* < 0.0001, and interactions between sex of participant-observer X sex of characteristic type, *X*^2^(1) = 17.02, *p* < 0.0001, sex of participant-observer X condition, *X*^2^(1) = 54.48, *p* < 0.0001, and sex of characteristic type X condition, *X*^2^(1) = 10.62, *p* = 0.0011. Overall, men (EMM = 0.177, SE = 0.013, 95%, CI [0.152, 0.202]) were more negative than women (EMM = 0.139, SE = 0.013, 95%, CI [0.117, 0.160]). Further, more negative reactions were observed for same-sex reactions (EMM = 0.237, SE = 0.009, 95% CI [0.219, 0.255]) than other-sex reactions (EMM = 0.159, SE = 0.007, 95% CI [0.145, 0.173]). Analysis of the sex of participant X sex of characteristic type interaction showed that for female advantageous characteristics, across conditions, women were more negative (EMM = 0.179, SE = 0.009, 95% CI [0.161, 0.196]) than men (EMM = 0.135, SE = 0.008, 95% CI [0.120, 0.150]), z = 4.825. *p* < 0.0001, while the difference for male advantageous characteristics was not significant between women (EMM = 0.231, SE = 0.011, 95% CI [0.211, 0.251]) and men (EMM = 0.246, SE = 0.011, 95% CI [0.225, 0.268]). Analysis of the sex of participant X condition interaction showed that while men were more negative overall with other-sex targets (EMM = 0.179, SE = 0.009, 95% CI [0.162, 0.197]) than women were (EMM = 0.138, SE = 0.008, 95% CI [0.123, 0.154]), z = 4.494, *p* < 0.0001, and women were more negative overall with same-sex targets (EMM = 0.271, SE = 0.011, 95% CI [0.249, 0.293]) than men were (EMM = 0.203, SE = 0.010, 95% CI [0.184, 0.222]), z = 6.493, *p* < 0.0001. In addition, women were significantly more negative with same-sex than with other-sex targets, z = 12.841, *p* < 0.0001, while this difference was not significant among men, z = 2.451, *p* = 0.068.

## Discussion

Are men more competitive than women? Answering this question is complicated by the fact that men and women pursue differing goals and employ different competitive strategies. In this study, we examined reported reactions to married women and men with children who vary in specific resources. Results of the present study show that participant-observers from three nations reported that based on their experiences, the women that they knew would respond more negatively to a same-sex target with a valuable characteristic than would men in the same situation. In other words, women react more negatively to resource asymmetry among same-sex peers than men do, but not to resource asymmetry with other-sex peers. In fact, participant-observers report that women are actually less negative than men are towards other-sex peers. Thus, it is not the case that women are more distressed than men by resource asymmetry in general, rather this difference is only present with same-sex peers. These results suggest that the motivation for competition over resources is greater in 25–45-year-old married women with children than in their male counterparts. This extends findings from previous studies that have found few self-reported sex differences in tendencies to compete over mates^28,29^ or observational studies suggesting when payoffs are high women may compete more than men^32,34^ to include women and men who are married with children and know others who differ in their physical and social assets.

The implications of the results for understanding human society are important in that they indicate that while women and men employ different competitive strategies and often pursue different goals, women may have an even greater motivation to compete with same-sex peers than men. Thus, it seems reasonable that women may be more envious than men of same-sex peers who are better able to care for their children. This is particularly pertinent when resources involved in competition directly impact same-sex individuals’ and their offspring’s survival. In contrast male-male competition is more likely to impact only sporadic mating behaviors^14,94^. Further study of competition between mothers seems warranted, as others have recommended^95,96^. Importantly, these results also demonstrate that women are more positive than men with respect to resource differentials in the other sex. Thus, from a man’s perspective, women are less competitive *with men* than men are, which might provide a further explanation for the often-cited conclusion that women are the less competitive sex. Although the more negative reactions to resource asymmetry could be interpreted as a form of inequity aversion, this would not change their role as a motivator of competition^97^. Nonetheless, it would be unlikely that inequity aversion would apply more towards one sex than the other.

Both women and men reported that resource differentials in characteristics that benefitted men would produce more negative reactions compared to characteristics that benefitted women. Although we did not predict this, it is possible that male advantageous characteristics are more consistent with classic measures of group status such as dominance and prestige^69^ or socioeconomic status^98^, which have been shown to be desired by and benefit both women and men in terms of survival^99^. Further, these group status characteristics that may be associated more with men than women may also provoke more negative reactions because universally men hold higher status than women^100^. More research is needed that focuses on characteristics that may be particularly beneficial to women^9^.

Limitations of our study include that we had participants from only three countries, and other countries may produce different results. Likewise, all of our participant-observers had access to computers which clearly is not representative of simple societies. A more diverse and larger sample would further buttress the validity of the findings. We also did not include participants or targets who did not identify as women or men, so we cannot generalize these results to non-binary individuals.

Finally, in this study we used a very simple definition of resource differential: possession of a single positive characteristic that has been shown to be beneficial and hence should be desired by others. Although more complex definitions abound, we feel that this nonetheless captures the essential component of competitiveness. Thus, to the extent that intolerance of resource differentials is a major driver of competitiveness, one that is independent of whatever strategy might be employed to attain the resource, our results suggest that at the least between 25 and 45 years of age, married women with children may be more competitive than their male counterparts. While this conclusion requires confirmation using different measures, these results provide a further challenge to the validity of traditional sexual selection theory in which human males are considered more competitive than females.

## Method

### Participants

For Study 1, we searched the online website Prolific (www.Prolific.org) for all countries that included at least 50 women and 50 men who were married with children and between the ages of 25 and 45 years who could serve as participant-observers. Three countries met the criteria: South Africa (SA), the United Kingdom (UK), and the United States (USA). We asked Prolific to invite 50 women and 50 men from each of these countries to become participant-observers. The numbers and mean ages of the participant-observers from each country were as follows: women (*M* = 32.82, *SE* = 0.65, n = 50) and men (*M* = 32.38, *SE* = 0.74, n = 48) in SA; women (*M* = 36.02, *SE* = 0.75, n = 50) and men (*M* = 37.69, *SE* = 0.73, n = 51) in the UK; and women (*M* = 36.22, *SE* = 0.67, n = 50) and men (*M* = 37.24, *SE* = 0.71, n = 50) in the USA.

For Study 2, we recruited 50 women and 50 men who were married with children and between the ages of 25 and 45 years from SA, the UK, and the USA to serve as participant-observers. None of the participants was included in Study 1. The numbers and mean ages of the participant-observers from each country were as follows: women (*M* = 31.49, *SE* = 0.80, n = 51) and men (*M* = 33.35, *SE* = 0.71, n = 49) in SA; women (*M* = 35.41, *SE* = 0.80, n = 49) and men (*M* = 36.42, *SE* = 0.66, n = 50) in the UK; and women (*M* = 36.51, *SE* = 0.76, n = 49) and men (*M* = 37.51, *SE* = 0.60, n = 49) in the USA.

### Procedure

In Study 1, interested female participants read a description of the study, agreed to become participant-observers, then read the following description:

Here are descriptions of 22 different target women who each possess a different positive characteristic. Each target woman is in her 30s and lives with her husband and children. The different types of positive characteristics these target women possess are resources, physical traits, status, and personal relationships.

We would be grateful for your honest opinion, based on your actual experience, about how most women without this characteristic would feel towards each target woman who has the characteristic. Assume that the women have seen the target woman in real life but do not know her personally.

For male participants, the description referred to men.

Each characteristic then was presented in random order after being preceded by the following question: “How would most (wo)men without this characteristic feel towards the target (wo)man who has the characteristic?” An example of one question to a female participant-observer based on a male advantageous characteristic would be “How would most women without this characteristic feel towards the target woman who has the characteristic: This woman holds a lot of power over others at her workplace, including making hiring, firing, and salary decisions.”

Participant-observers then indicated whether most same-sex individuals would feel “Quite Positively,” “Somewhat Positively,” “Somewhat Negatively,” or “Quite Negatively” towards every target individual. Since we were specifically interested in tendencies towards negative evaluations, responses were coded as binary, with “1” recorded if the response included either of the two negative evaluations or else “0.” In order to insure that this method of coding responses was accurate, the analyses were redone using the full continuous scale. These are included in the SOM, and give the same basic results. 22 different target individuals were described to reduce the probability that participant-observers would assume that the same target possessed several different positive characteristics.

Study 2 was identical to Study 1, except that the sex of the target was switched. Female participants were asked to indicate how women would respond to a male target who possessed a positive characteristic that they did not, and male participants were asked to indicate how men would respond to a female target who possessed a positive characteristic that they did not. In all cases, the 22 other-sex targets were described as married men or women who were in their 30 s and had children.

### Ethics statement

The study was approved by the Research Ethics Committee of the University of Quebec at Montreal. The methods used were performed in accordance with relevant guidelines.

### Informed consent

Informed consent was obtained from all participants.

## Supplementary Information

Supplementary Information.

## Data Availability

All data and R scripts are available at https://osf.io/df964/.
