# Supplementary material for: Married women with children experience greater intrasexual competition than their male counterparts
Source: Sci Rep. 2023 Mar 18;13:4498. doi: 10.1038/s41598-023-31816-0 (PMC10024730; doi:10.1038/s41598-023-31816-0)
Supplement: Supplementary file 1 — Supplementary Information. [file 41598_2023_31816_MOESM1_ESM.docx]

Analysis of evaluations using a linear mixed model (LMM) with mean response as dependent variable with sex of characteristic type (female advantageous versus male advantageous) as a repeated measure, sex of participant-observer and condition (same-sex versus other-sex target) as independent variables, with country as a random effect, and age and age of oldest child as covariates.

Study 1

For Study 1 which examined reactions to same-sex targets who possessed a positive characteristic that most same-sex individuals did not have, results yielded significant effects of sex of participant-observer, *X*^2^(1) = 24.21, *p*<.0001 and sex of characteristic type, *X*^2^(1) = 32.64, *p*<.0001. No effect of age nor of age of oldest child was found. Significantly female participant-observers reported that women lacking a characteristic would feel more negatively towards women with the characteristic (EMM = 8.05, SE = .775, 95% CI [6.07, 10.00]) than male participant-observers reported for men’s reactions towards a man possessing the characteristic (EMM = 11.31, SE = .785, 95% CI [9.32, 13.3]). Additionally, both men and women participant-observers reported more negative reactions for male advantageous characteristics (EMM = 7.82, SE = .781, 95% CI [5.81, 9.82]) than for female advantageous characteristics (EMM = 11.55, SE = 776, 95% CI [9.56, 13.54]).

Study 2

Results from Study 2 yielded significant effects of sex of participant-observer, *X*^2^(1) = 24.05, *p*<.0001, sex of characteristic type, *X*^2^(1) = 101.77, *p*<.0001, their interaction, *X*^2^(1) = 9.08, *p*=.003. In marked contrast to Study 1, female participant-observers reported that women lacking a characteristic would feel less negatively towards men with the characteristic (EMM = 14.1, SE = 1.04, 95% CI [10.80, 17.4]) than male participant-observers reported for men’s reactions towards women possessing the characteristic (EMM = 11.2, SE = 1.04, 95% CI [7.96, 14.50]). As in Study 1, participant-observers reported more negative evaluations for male advantageous characteristics (EMM = 9.82, SE = 1.04, 95% CI [6.55, 13.10]) than for female advantageous characteristics (EMM = 15.51, SE = 1.03, 95% CI [12.23, 18.80]). Contrast analyses on the interaction showed that the difference between women’s and men’s negative reactions were significant only for male advantageous characteristics (Male: (EMM = 7.54, SE = 1.11, 95% CI [4.04, 11.10]), Female: (EMM = 12.10, SE = 1.12, 95%, CI [8.62, 15.6]), *t* = 5.605, *p* = .033), not for female advantageous characteristics (Male: (EMM = 14.93, SE = 1.11, 95% CI [11.42, 18.40]), Female: (EMM = 16.09, SE = 1.11, 95%, CI [12.57, 19.60]), *t = 1.443, p = .556*).

Combined analyses

As a final analysis, we combined the results of both studies, and performed a linear mixed model (LMM) with mean response as dependent variable sex of characteristic type (female advantageous versus male advantageous) as a repeated measure, sex of participant-observer and condition (same-sex versus other-sex target) as independent variables, with country as a random effect, and age and age of oldest child as covariates. Results yielded significant effects of sex of characteristic type, *X*^2^(1) = 116.18, *p*<.0001, condition, *X*^2^(1) = 46.79, *p*<.0001, and interactions between sex of participant-observer X condition, *X*^2^(1) = 48.13, *p*<.0001, and sex of characteristic type X condition, *X*^2^(1) = 4.89, *p=*.0271. Reactions were more negative with same-sex evaluations (EMM = 9.71, SE = .309, 95% CI [9.09, 10.3]) than other-sex evaluations (EMM = 12.64, SE = .310, 95% CI [12.03, 13.3]). Additionally, participant-observers reported more negative reactions for male advantageous characteristics (EMM = 8.82, SE = .312, 95% CI [8.2, 9.44]) than for female advantageous characteristics (EMM = 13.53, SE = .307, 95% CI [12.9, 14.14]) for both men *and* women. Analysis of the sex of participant X condition interaction showed that for other-sex targets men were more negative (EMM = 11.23, SE = .439, 95% CI [10.36, 1210]) than women were (EMM = 14.05, SE = .443, 95% CI [13.18, 14.93]), t=4.503, p=0001, while women were more negative with same-sex targets (EMM = 8.06, SE = .430, 95% CI [7.21, 8.92]) than men were (EMM = 11.35, SE = .447, 95% CI [10.46, 12.24]), t=5.295, p<.0001. In addition, women were significantly more negative with same-sex than with other-sex targets, t=9.737, p<.0001, while this difference was not significant among men, t<1. Analysis of the sex of characteristic type X condition interaction showed that male advantageous characteristics were rated more negatively than female advantageous characteristics, they were both rated significantly more negatively with same-sex targets than with other-sex targets.
